# Carcinoembryonic Antigen: A Potential Biomarker to Evaluate the Severity and Prognosis of COVID-19

**DOI:** 10.3389/fmed.2020.579543

**Published:** 2020-10-06

**Authors:** Qianqian Chen, Hui Kong, Xu Qi, Wenqiu Ding, Ningfei Ji, Chaojie Wu, Chaolin Huang, Wenjuan Wu, Mao Huang, Weiping Xie, Yun Liu, Jinhai Tang

**Affiliations:** ^1^Department of Respiratory and Critical Care Medicine, The First Affiliated Hospital of Nanjing Medical University, Nanjing, China; ^2^Division of Intensive Care Unit, Wuhan Jin Yin-tan Hospital, Wuhan, China; ^3^Department of Medical Informatics, School of Biomedical Engineering and Informatics, Nanjing Medical University, Nanjing, China; ^4^Department of General Surgery, The First Affiliated Hospital of Nanjing Medical University, Nanjing, China

**Keywords:** corona virus disease 2019, severe acute respiratory coronavirus 2, carcinoembryonic antigen, biomarker, prognosis

## Abstract

**Background and Objectives:** Corona Virus Disease 2019 (COVID-19) has become a serious pandemic disease worldwide. Identification of biomarkers to predict severity and prognosis is urgently needed for early medical intervention due to high mortality of critical cases with COVID-19. This retrospective study aimed to indicate the values of carcinoembryonic antigen (CEA) in evaluating the severity and prognosis of COVID-19.

**Methods:** We included 46 death cases from intensive care unit and 68 discharged cases from ordinary units with confirmed COVID-19 of Wuhan Jin Yin-tan Hospital from January 1 to March 22, 2020. Laboratory and radiologic data were analyzed retrospectively. All patients were followed up until April 10, 2020.

**Results:** COVID-19 patients in the death group had significantly higher CEA levels (ng/ml) than discharged group (14.80 ± 14.20 vs. 3.80 ± 2.43, *P* < 0.001). The risk of COVID-19 death increased 1.317 times for each additional 1 ng/ml CEA level (OR = 1.317, 95% CI: 1.099–1.579). The standardized and weighted receiver operating characteristic curve (ROC) analysis adjusted to age, sex, and ferritin levels suggested that the area under the curve (AUC) of the serum CEA levels was 0.808 in discrimination between death cases and discharged cases with COVID-19 (*P* < 0.001). We found mortality of COVID-19 is associated with elevated CEA levels increased (HR = 1.023, 95% CI: 1.005–1.042), as well as age (HR = 1.050, 95% CI: 1.016–1.086) and ferritin levels (HR = 1.001, 95% CI: 1.001–1.002) by survival analysis of Cox regression model. Among discharged patients, CEA levels were significant lower in moderate cases compared to the severe and critical cases (*P* = 0.005; OR = 0.488, 95% CI: 0.294–0.808) from binary logistic regression analysis. The AUC of CEA levels was 0.79 in distinguishing moderate cases from discharged COVID-19 patients by standardized and weighted ROC analysis (*P* < 0.001). A positive correlation between CEA levels and CT scores existed in discharged patients (Correlation Coefficient: 0.687; *P* < 0.001).

**Conclusions:** Elevated CEA levels increased the risk of death from COVID-19 and CEA levels were related to CT scores of the discharged patients positively.

## Introduction

Corona Virus Disease 2019 (COVID-19) is a disease caused by severe acute respiratory coronavirus 2 (SARS-CoV-2) (1). It has developed into a serious pandemic disease worldwide and become a burden borne by health care systems in many countries since its first outbreak in Wuhan, China in December, 2019 (2, 3). According to the latest COVID-2019 situation reports released by World Health Organization (WHO) (4), there were over 23 million patients with confirmed COVID-19 globally, along with over 806,000 death cases by August 24, 2020. It was reported that the case-fatality rates of critical cases were about 24-fold higher than those of confirmed cases with COVID-19 (49.0 vs. 2.3%) (5). In view of high mortality of critical cases, identifying specific biomarkers for estimate severity and prognosis of COVID-19 is in urgent need, especially when local health systems are overwhelmed. A few inflammatory factors, coagulation parameters or cytokines were considered as potential biomarkers of COVID-19 progression or severity (6–9). However, it is a long way to identify a simple biomarker with higher sensitivity or sensitivity for better comprehensive evaluation.

Carcinoembryonic antigen (CEA), also known as CEA-related cell adhesion molecule 5 (CEACAM5) or CD66e, is a kind of glycophosphatidylinositol-linked membrane glycoprotein anchored in a specific cell membrane microdomains (10). As a cell adhesion molecule, CEA functioned as a bridge linked to pathogens or stromal cells with other members of epithelial CEACAMs, triggers CEACAM-mediated signal communication and activated integrin signaling pathways in human endothelial cells (10–12). CEA is not only related to respiratory or digestive cancers but also some infectious diseases like gonorrhea or chronic inflammatory diseases like interstitial lung diseases (ILD) (13, 14). Immunohistochemical analysis of lung specimens from patients with pulmonary fibrosis indicated that strong expression of CEA in metaplastic bronchiolar and type II alveolar epithelia (15, 16). Formation of abnormal epithelial proliferation and renewal was seen as a possible source to elevated CEA levels in patients with pulmonary fibrosis (15). Similar to pathological changes of ILD, significant hyperplasia of type II alveolar epithelial cells and interstitial fibrosis were referred to from several reports of COVID-19 autopsies and biopsies (17, 18). To our knowledge, only one study about the serum cancer biomarkers including CEA levels of COVID-19 patients have been published (19). Whether elevated CEA levels are associated with poor prognosis or severity of COVID-19 remains unclear nevertheless.

Therefore, we conducted this study to present the serum CEA levels of COVID-19 patients and indicate the relation between serum CEA levels and prognosis or severity of COVID-19, which may be of great value for effective treatment.

## Materials and Methods

### Data Sources and Extraction

A total of 46 death cases with confirmed COVID-19 were included in our study, which were admitted to an intensive care unit (ICU) for COVID-19 of Wuhan Jin Yin-tan Hospital from January 1 to March 22, 2020. Respectively, 68 discharged patients (13 from ICU, 55 from ordinary units) were included during the same period. All medical records of the patients we included were reviewed and related raw data were collected by the two authors independently. A senior investigator reviewed the raw data if there were any discrepancies. The following data of each patient we included were extracted: age, sex, smoking history, length of stay, comorbidities, white blood cell count, white blood cell count, lymphocyte count, neutrophil count, monocyte count, C-reactive protein (CRP), ferritin, interleukin 6 (IL-6), alpha fetoprotein (AFP), and CEA; radiology data including chest computed tomography (CT) or chest X-rays (for patients in ICU). Comorbidities of all patients were collected on admission. We only included the baseline data on admission if patients have the same kind of laboratory tests or radiology examination more than once. The survival time were defined as the time (days) from admission date to observation endpoint (death or April 10, 2020). Our study was approved by the Ethics Committee of Wuhan Jin Yin-tan Hospital (No. KY-2020-59.01).

### Inclusion and Exclusion Criteria

All patients included met the following inclusion criteria: Confirmed COVID-19 cases was diagnosed by real-time fluorescent reverse transcription-polymerase chain reaction (RT-PCR) based on “Diagnosis and Treatment Protocol for Novel Coronavirus Pneumonia (Trial Version 7)” published by the National Health Commission of the People's Republic of China (20). Exclusion criteria were followed below: (1) Patients with a history of any cancer or any chronic disease related to elevated CEA levels like chronic kidney disease. (2) Patients infected with other viruses or bacteria.

### Clinical Classification of COVID-19

The clinical classification of COVID-19 in our study was based on “Diagnosis and Treatment Protocol for Novel Coronavirus Pneumonia (Trial Version 7)” published by the National Health Commission of the People's Republic of China (20). Patients with COVID-19 are divided into four types: mild cases, moderate cases, severe cases, and critical cases (detailed descriptions on Supplementary Material).

### Radiological Assessment by CT Scores

The CT scans of all discharged COVID-19 patients were assessed by two radiologists with more than 10 years of experience in a semi-quantitative way established by Xie et al. (21). Details of the criteria for the CT scores can be found on Supplementary Material. The definitions of lung lesions were specified in another study (22).

### Statistical Analysis

Continuous variables were analyzed by Student's *t*-test while categorical variables were analyzed by Chi-square test. Binary logistic regression was applied for analysis of survival parameters. The variables with *P*-values < 0.05 in the univariate analysis were included in the binary logistic regression. The detailed descriptions of the model development and evaluation were mentioned in the previous study (23). We applied Nagelkerke *R*^2^ to measure overall model performance and the area under the receiver operating characteristic curve (AUC) to evaluate discriminative ability of each model (24). Hosmer–Lemeshow Test was used for Goodness of Fit Test. Only one inflammatory factor and one tumor marker were included in the same logistic model to avoid multicollinearity of interactive variables. We used standard and weighted receiver operating characteristic (ROC) curve analysis to assess the sensitivity and specificity of different parameters as biomarkers of COVID-19 prognosis or severity. The weighted ROC curve analysis was defined and performed as descriptions in a published article by the way of inverse probability weighting (25). The survival analysis was conducted by Cox regression model (proportional hazard model) and Kaplan–Meier analysis with Mantel–Cox Test. The relation between CEA levels and other factors in discharged cases was evaluated by Spearman's correlation coefficient. *P*-values < 0.05 in our study were statistically significant. We performed statistical analysis with IBM SPSS Statistics (Version 24.0).

## Results

### Demographic and Clinical Characteristics of the Patients

According to the inclusion and exclusion criteria, we totally included 114 patients with COVID-19 (46 death cases and 68 discharged cases) in our study, with two patients with cancers, one with chronic kidney disease, and one without CEA levels excluded. All demographic and clinical characteristics of the patients we included were demonstrated in Table 1. The mean age of all patients included was 61.74 ± 13.57. The patients in death group were older than those in discharged group (65.93 ± 8.49 vs. 58.60 ± 15.47, *P* = 0.002). There were 15 females in death group, which were less than those in discharged group (32.61 vs. 55.88%, *P* = 0.015). Death cases spent shorter time in hospital than discharged cases (11.48 ± 8.79 vs. 25.28 ± 17.34, *P* < 0.001). In complete blood count, white blood cell count (14.21 ± 8.18 vs. 5.87 ± 2.70, *P* < 0.001) and neutrophil count (13.04 ± 8.51 vs. 4.09 ± 2.66, *P* = 0.001) was increased significantly in death cases with COVID-19, while lymphocyte count decreased significantly (0.77 ± 0.35 vs. 1.25 ± 0.59, *P* < 0.001). We found CRP (*P* < 0.001) and ferritin levels (*P* < 0.001) were significantly higher in the death group than discharged group among inflammation indicators included in our study. No differences were found in IL-6 levels between the two groups (*P* = 0.381). Compared with the discharged group, the serum CEA levels were higher in the death group (14.80 ± 14.20 vs. 3.80 ± 2.43, *P* < 0.001).

**Table 1:** Demographic and clinical characteristics of the COVID-19 patients included.

	**Total (*n* = 114)**	**Death group (*n* = 46)**	**Discharged group (*n* = 68)**	***P*-values**
Age (years)	61.74 ± 13.57	65.93 ± 8.49	58.60 ± 15.47	0.002
Sex				0.015
Female %	53 (46.50)	15 (32.61)	38 (55.88)	
Male %	61 (53.50)	31 (67.39)	30 (44.12)	
Smoker %	45 (39.47)	23 (50.00)	22 (32.35)	0.059
Comorbidities %	65 (57.02)	30 (65.22)	35 (51.47)	0.146
Diabetes mellitus	27 (23.68)	14 (30.43)	13 (19.12)	0.163
Cardiovascular disease	12 (10.53)	3 (6.52)	9 (13.24)	0.252
COPD	5 (4.39)	3(6.52)	2(2.94)	0.360
Hypertension	50(43.86)	23(50.00)	27(39.71)	0.277
Length of stay (days)	19.56 ± 15.91	11.48 ± 8.79	25.28 ± 17.34	<0.001
White blood cell count (× 10^9^/L)	7.90 ± 5.80	14.21 ± 8.18	5.87 ± 2.70	<0.001
Lymphocyte count (× 10^9^/L)	1.15 ± 0.57	0.77 ± 0.35	1.25 ± 0.59	<0.001
Neutrophil count (× 10^9^/L)	6.22 ± 5.98	13.04 ± 8.51	4.09 ± 2.66	0.001
Monocyte count (× 10^9^/L)	0.42 ± 0.20	0.44 ± 0.23	0.41 ± 0.19	0.766
C-reactive protein (mg/L)	44.58 ± 54.27	122.96 ± 62.13	28.44 ± 38.48	<0.001
Ferritin (ng/ml)	792.78 ± 684.73	1315.06 ± 653.17	423.81 ± 423.79	<0.001
Alpha fetoprotein (ng/ml)	2.48 ± 1.64	1.75 ± 0.85	2.98 ± 1.84	<0.001
Carcinoembryonic antigen (ng/ml)	8.23 ± 10.64	14.80 ± 14.20	3.80 ± 2.43	<0.001
Interleukin 6 (pg/ml)	22.30 ± 39.59	41.95 ± 40.11	20.10 ± 39.66	0.381

*COPD, chronic obstructive pulmonary disease. Continuous variables are showed as mean ± SD*.

### Association Between CEA Levels and COVID-19 Prognosis

**Figure 1:**
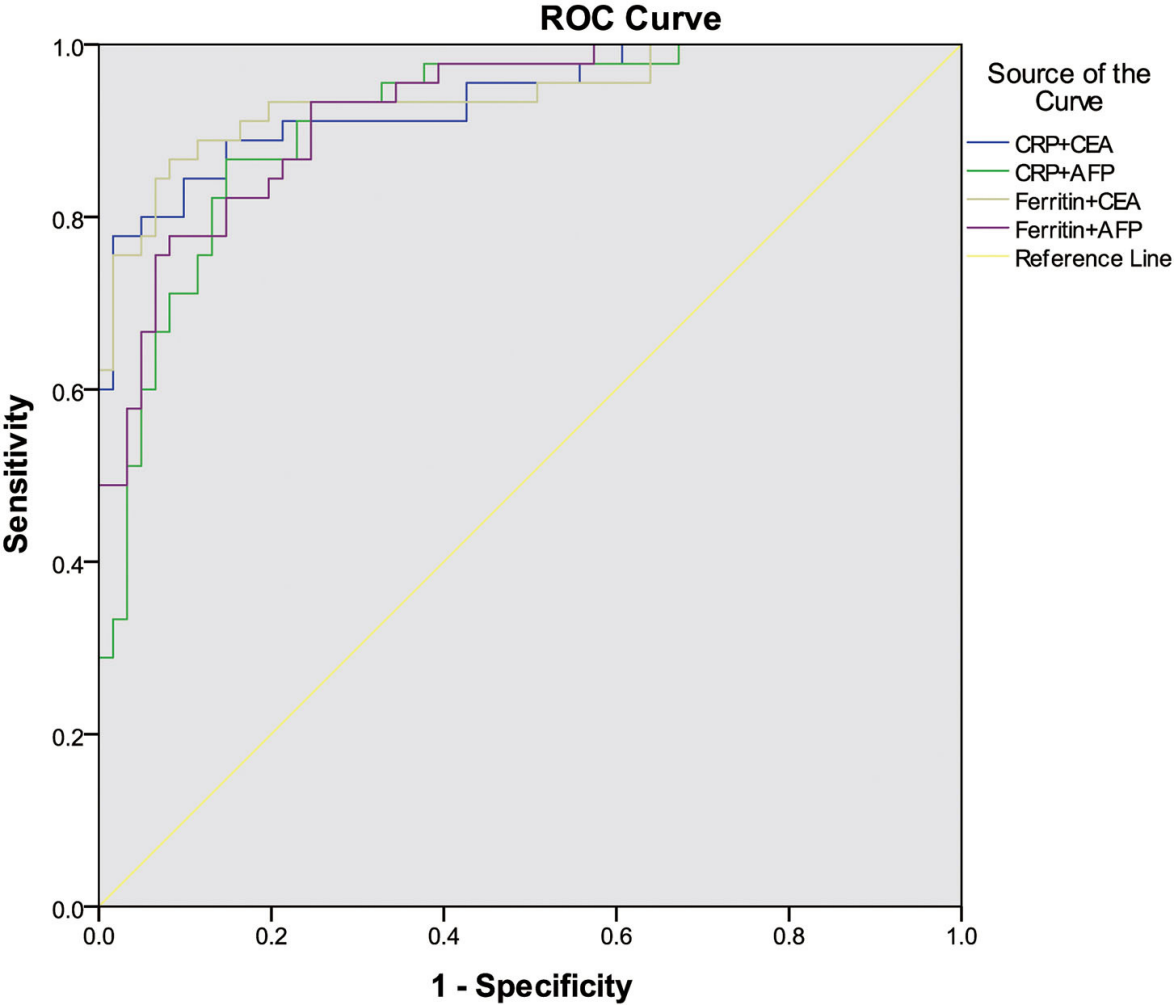
The ROC curve analysis of different binary logistic regression models for the prediction of COVID-19 prognosis.

**Figure 2:**
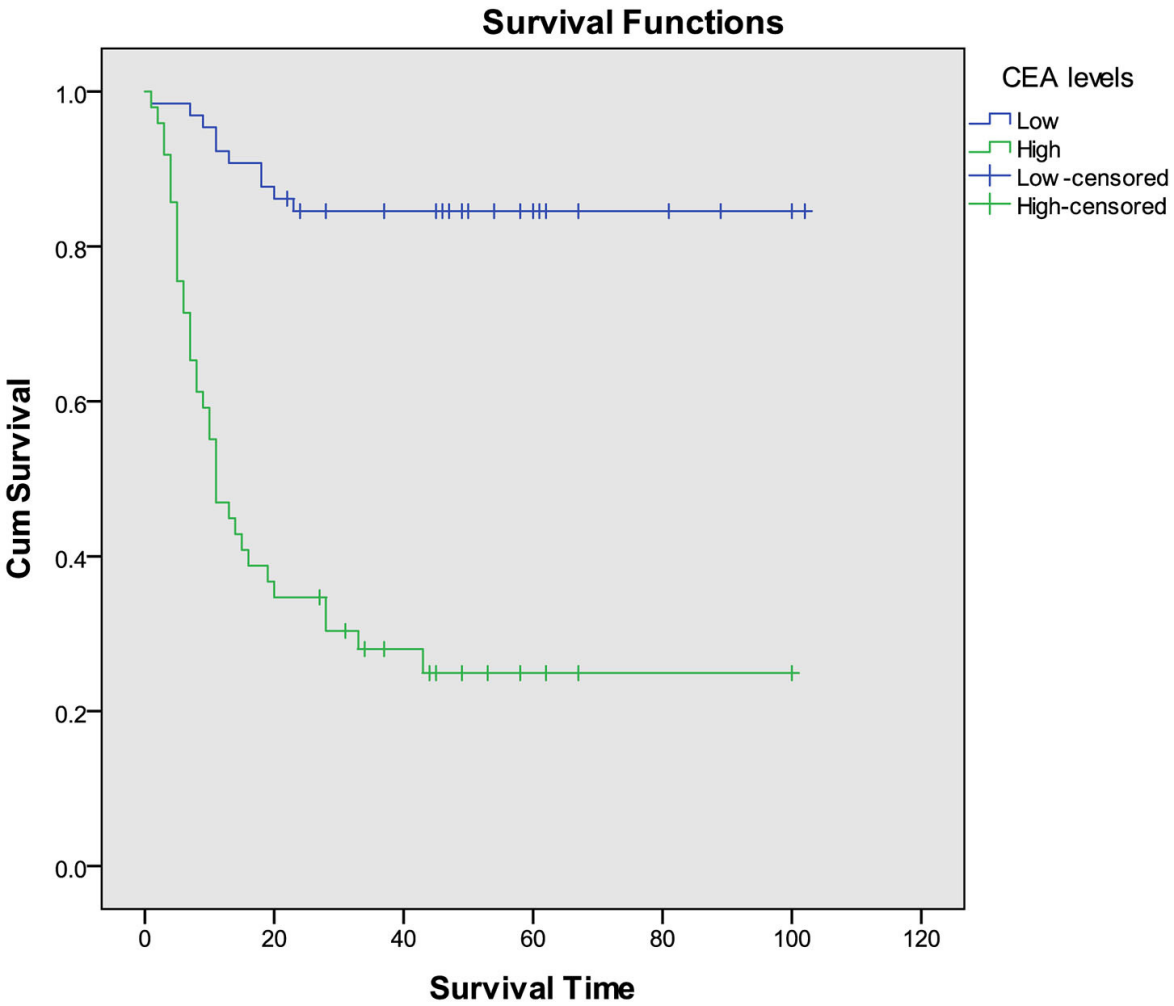
Kaplan–Meier survival analysis of COVID-19 patients with different CEA levels.

For multivariate analysis, we used binary logistic regression to build four models (Table 2). Variables with *P*-values < 0.05 in univariate analysis were included in our logistic regression models including demographic factors (sex, age, and smoking history), inflammatory factors (CRP or ferritin), and tumor markers (CEA or AFP). We found Model 3 including age, sex, smoking history, ferritin levels, and CEA levels showed the most discriminative ability among the four models we built in Figure 1 (Nagelkerke *R*^2^ = 0.729; AUC = 0.940). The Goodness of Fit Test of Model 3 was 0.408 by Hosmer–Lemeshow Test. From the binary logistic regression analysis of Model 3, age (*P* = 0.009), sex (*P* = 0.041), ferritin levels (*P* < 0.001) and CEA levels (*P* = 0.003) were significantly related to the prognosis of COVID-19. After adjustment to age, sex, and smoking history, the binary logistic regression equation (Model 3) demonstrated that the risk of COVID-19 death increased 1.002 times for each additional 1 mg/L ferritin level (OR = 1.002, 95% CI: 1.001–1.003) and 1.317 times for each additional 1 ng/ml CEA level (OR = 1.317, 95% CI: 1.099–1.579). The ROC curve analysis suggested that AUC of the serum CEA levels was 0.808 by standardized and weighted ROC curve analysis adjusted to other cofounding factors (age, sex, and ferritin levels). From survival analysis of proportional hazard model, we also found elevated CEA levels increased mortality of COVID-19 (HR = 1.023, 95% CI: 1.005–1.042), as well as age (HR = 1.050, 95% CI: 1.016–1.086) and ferritin levels (HR = 1.001, 95% CI: 1.001–1.002). The COVID-19 patients were divided into two groups (high CEA levels group and low CEA levels group) based on the cutoff value of CEA levels (5.2 ng/ml) calculated by time-dependent ROC curve. The COVID-19 patients with high CEA levels had higher risk of death than those with low CEA levels by Kaplan–Meier survival analysis (Figure 2). The Mantel–Cox Test of Kaplan–Meier survival analysis was *P* < 0.001.

**Table 2:** Binary logistic regression analysis of factors related to covid-19 prognosis.

**Variable**		**Model**			
		**1**	**2**	**3**	**4**
Demographic factors	Age	1.072 (1.000–1.149)	1.062 (1.007–1.121)	1.104 (1.025–1.188)	1.057 (1.002–1.115)
	Sex	0.178 (0.029–1.113)	0.255 (0.048–1.347)	0.128 (0.018–0.918)	0.249 (0.048–1.284)
	Smoker	0.777 (0.146–4.125)	0.358 (0.070–1.838)	0.522 (0.088–3.100)	0.335 (0.066–1.706)
Inflammatory factors	CRP	1.023 (1.012–1.034)	1.024 (1.013–1.034)	-	-
	Ferritin	-	-	1.002 (1.001–1.003)	1.003 (1.002–1.004)
Tumor markers	CEA	1.311 (1.096–1.568)	-	1.317 (1.099–1.579)	-
	AFP	-	0.489 (0.272–0.879)	-	0.503 (0.281–0.899)
Model evaluation	Nagelkerke R^2^	0.724	0.620	0.729	0.655
	AUC	0.936	0.914	0.940	0.924

*Variables in the model are showed as OR (95% CI)*.

### Relation Between CEA Levels and COVID-19 Severity in Discharged Patients

**Figure 3:**
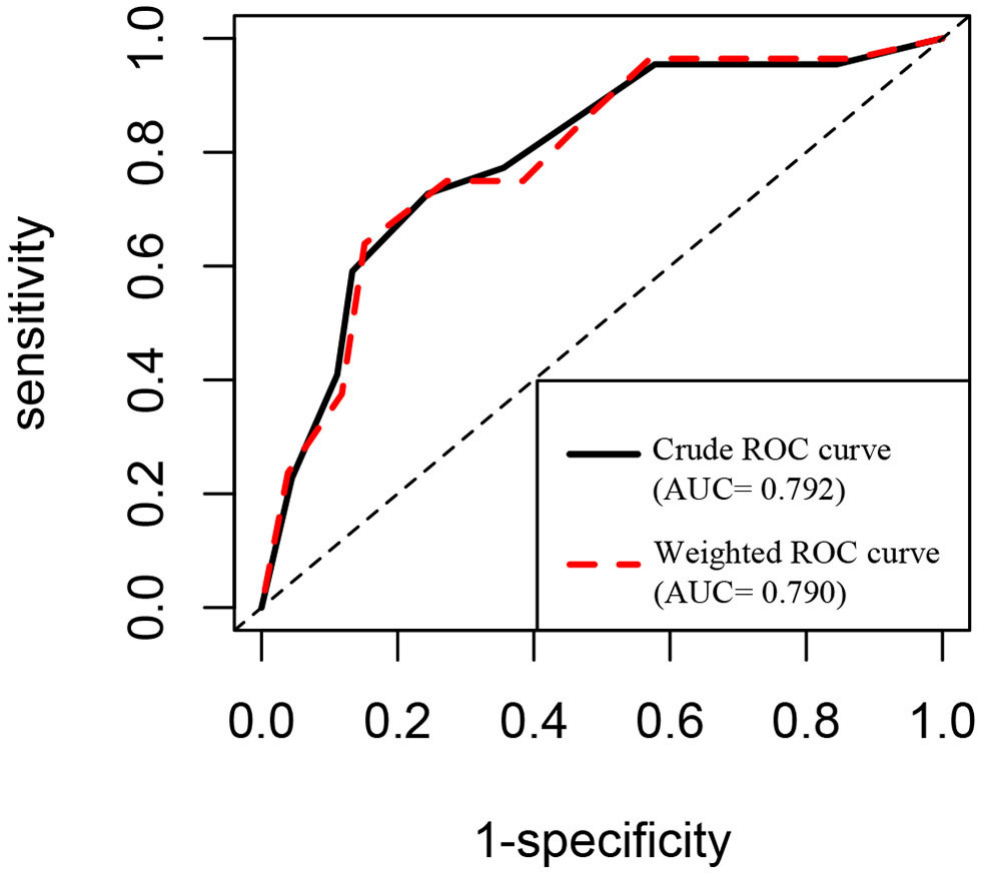
ROC curve analysis of CEA levels in distinguishing moderate cases from discharged COVID-19 patients.

**Figure 4:**
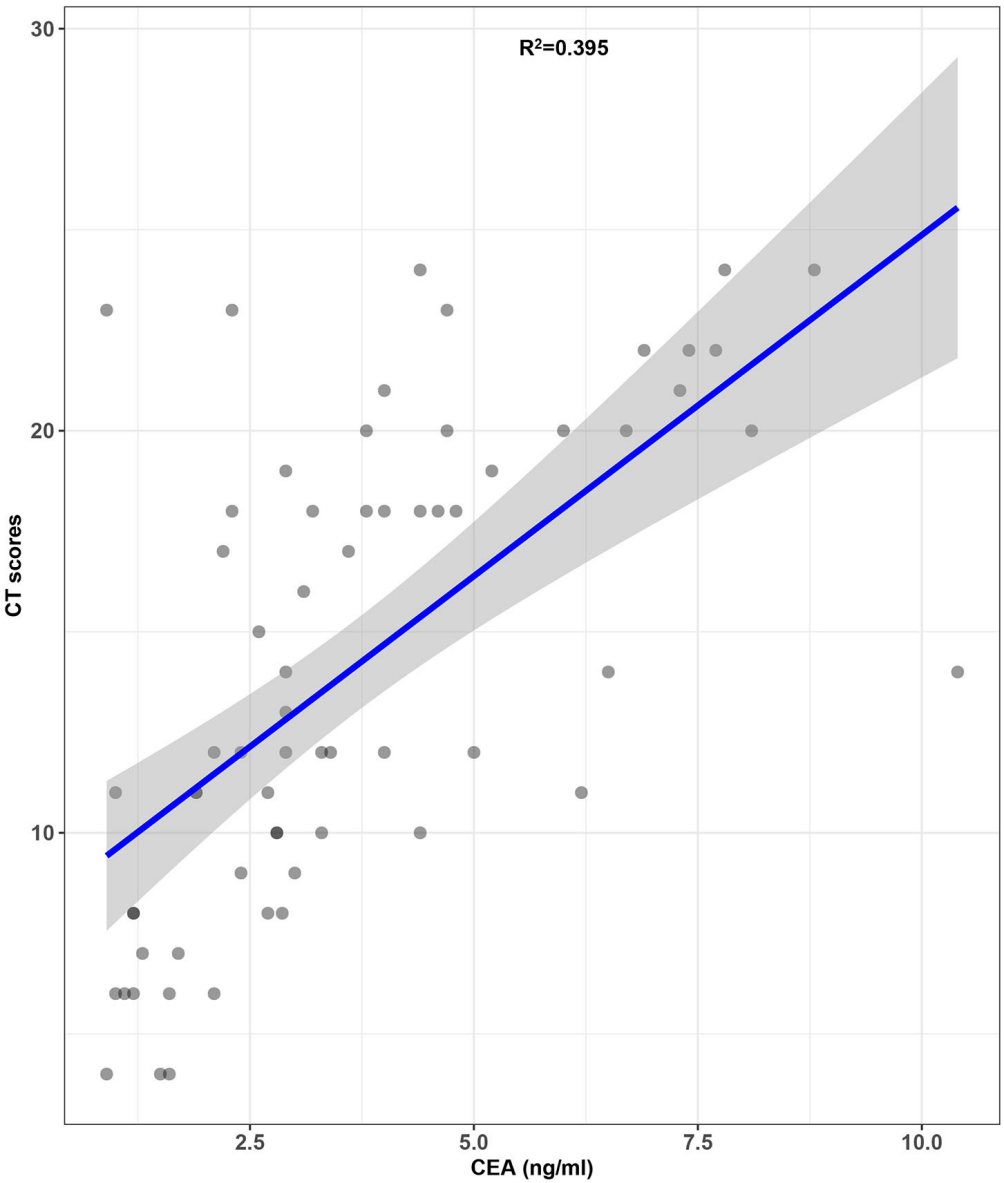
Correlation of CEA levels with CT scores in discharged COVID-19 patients (the gray area on the figure means 95% confidence intervals).

**Figure 5:**
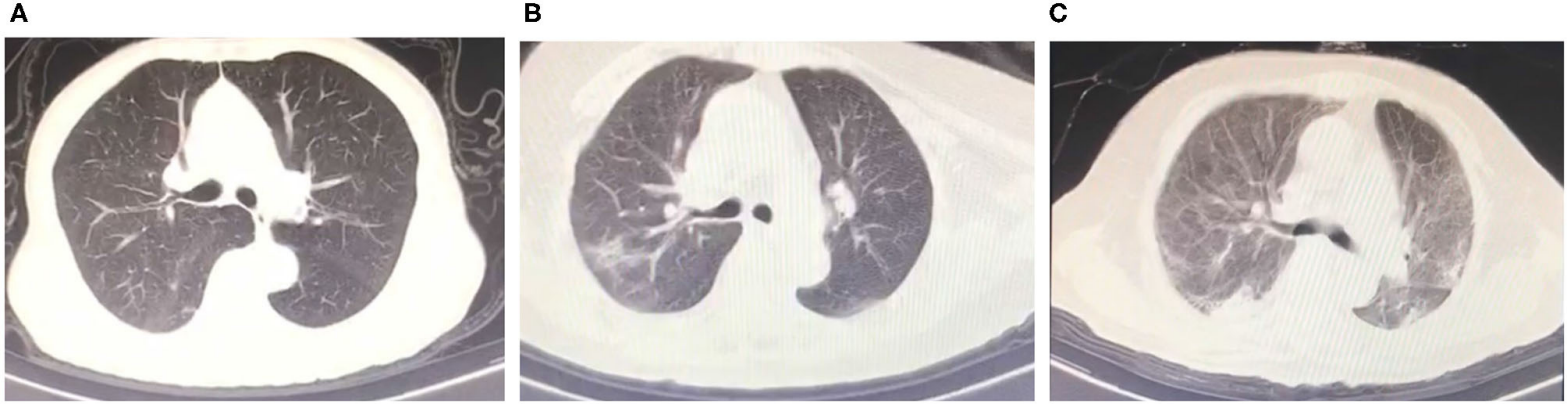
Chest CT scans and CEA levels of three female patients. **(A)** Four points CT scores and 0.9 ng/ml CEA levels. (70 years old). **(B)** Sixteen points CT scores and 3.1 ng/ml CEA levels (70 years old). **(C)** Twenty two points CT scores and 7.7 ng/ml CEA levels (69 years old).

A significant difference in CEA levels (*P* < 0.001) was found between the discharged patients with different clinical classifications, while age (*P* = 0.001) and other laboratory test including white blood cell count (*P* = 0.008), neutrophil count (*P* = 0.012), monocyte count (*P* = 0.031) and ferritin levels (*P* = 0.039) in Table 3. The results of binary logistic regression analysis (Table 4) revealed that CEA levels were lower (*P* = 0.005; OR = 0.488, 95% CI: 0.294–0.808) in moderate cases compared with the severe and critical cases after adjusted to age and the laboratory tests, which were statistic significantly in the univariate analysis. At the same time, moderate COVID-19 patients were younger than severe and critical cases from binary logistic regression analysis (*P* = 0.039; OR = 0.858, 95% CI: 0.742–0.993). Figure 3 shows the ROC curves of CEA levels with the AUC of 0.790 (*P* < 0.001) in distinguishing moderate cases from discharged COVID-19 patients after considering age as a cofounding factor by weighted ROC analysis. The results of Spearman's correlation analyses (Figure 4) indicated that there was a positive correlation of CEA levels with CT scores in discharged patients (Correlation Coefficient: 0.687; *P* < 0.001). The Figure 5 showed chest CT scans and CEA levels of three female patients in similar old age (69, 70, 71).

**Table 3:** Characteristics of 68 discharged patients with different disease severities.

	**Moderate (*n* = 46)**	**Severe/critical (*n* = 22)**	***P*-values**
Age (years)	54.85 ± 15.78	66.95 ± 11.00	0.001
Sex			0.115
Female %	22 (47.83)	15 (68.18)	
Male %	24 (52.17)	7 (31.82)	
Smoker %	15 (32.61)	7 (31.82)	0.948
Comorbidities %	25 (54.35)	10 (45.45)	0.669
Diabetes mellitus	10 (21.74)	3 (13.64)	0.642
Cardiovascular disease	4 (8.70)	5 (22.73)	0.224
COPD	0 (0.00)	2 (9.10)	0.191
Hypertension	18 (39.13)	9 (40.91)	0.889
White blood cell count (× 10^9^/L)	5.31 ± 2.45	7.19 ± 2.94	0.008
Lymphocyte count (× 10^9^/L)	1.28 ± 0.58	1.17 ± 0.60	0.466
Neutrophil count (× 10^9^/L)	3.54 ± 2.27	5.80 ± 3.12	0.012
Monocyte count (× 10^9^/L)	0.39 ± 0.17	0.53 ± 0.27	0.031
CRP (mg/L)	27.53 ± 39.57	35.96 ± 37.14	0.424
Ferritin (ng/ml)	353.55 ± 295.24	578.94 ± 583.51	0.039
AFP (ng/ml)	2.96 ± 1.45	3.03 ± 2.51	0.895
CEA (ng/ml)	3.04 ± 1.97	5.35 ± 2.58	<0.001
IL-6 (pg/ml)	23.83 ± 44.05	10.72 ± 5.93	0.333
CT scores	12.57 ± 5.51	17.38 ± 5.53	0.002

*COPD, chronic obstructive pulmonary disease. Continuous variables are showed as mean ± SD*.

**Table 4:** Binary logistic regression analysis of factors related to COVID-19 severities.

**Valuable**	***P*-value**	**OR**	**95% CI**
Age	0.039	0.858	0.742–0.993
White blood cell count	0.833	1.246	0.161–9.662
Neutrophil count	0.729	0.708	0.100–5.005
Monocyte count	0.465	0.032	0.000–31.614
Ferritin	0.116	0.997	0.994–1.001
CEA	0.005	0.488	0.294–0.808
Constant	0.007	-	-

## Discussion

In our study, we found the serum CEA levels increased in non-survivors with COVID-19 compared to discharged patients statistically. Moreover, increasing levels of serum CEA were associated with more CT involvement scores in discharged COVID-19 patients. Till now, only one study reported the elevated serum CEA levels in COVID-19 patients (19). In that retrospective study, the authors found significant increases of CEA levels compared with health controls and a potential association between CEA and CRP levels. However, the role of CEA levels in clinical outcomes or CT involvements of COVID-19 patients was still unknown. This study validated the previous result that CEA levels were related to severity of COVID-19 reported by different research groups (Union Hospital of Tongji Medical College, Wuhan) (19). Furthermore, our study concentrated on different CEA levels in COVID-19 patients with different clinical outcomes (survivors or non-survivors) and potential correlations with CT involvement scores. We firstly found CEA levels are associated with prognosis and severity of COVID-19. We also calculated the value of CEA levels in evaluating the severity and prognosis of COVID-19.

Some hypotheses were formulated to explain the correlation between elevated CEA levels and poor prognosis or severity of COVID-19. Generally, CEA is considered to be a significant biomarker for adenocarcinoma in respiratory system or digestive system. Additionally, it should be noted that serum CEA levels were also significantly increased in some non-neoplastic lung diseases, especially in ILD or allergic bronchopulmonary aspergillosis (ABPA). In respiratory system, increased CEA expression of bronchiolar cells and type II pneumocytes (15) was responded to inflammation induced by mucus plug in ABPA (26) or atypical epithelial proliferation in idiopathic pulmonary fibrosis (IPF) (16). As human angiotensin-converting enzyme 2 (ACE2) is an entry receptor of SARS-CoV-2 (27, 28), bronchiolar epithelial cells and type II alveolar epithelial cells are the prime targets of SARS-CoV-2 in lung because of abundant expression on lung alveolar epithelial cells where SARS-CoV-2 particles were observed by electron microscopy (29, 30). Several autopsy studies have confirmed that bronchi were covered by mucus or mucus plug, as well as significant proliferating interstitial fibroblasts and type II pneumocyte hyperplasia (31) in COVID-19 patients. Since type II pneumocytes are considered as the stem cells or progenitors in alveoli (32), SARS-CoV-2 infection-induced massive alveolar epithelial cell death may lead to abnormal regeneration of type II pneumocytes for repair along with the production of CEA, which was similar to the uncontrolled proliferation of lung adenocarcinoma. Moreover, atypical epithelial proliferation and proliferating fibroblasts may also exacerbate obstruction of bronchioles and lung consolidation leading to refractory hypoxemia as well as worsen CT involvement scores. Therefore, it is reasonable that serum CEA levels correlate with the severity and prognosis of COVID-19.

Based on the relationship between CEA and type II pneumocyte hyperplasia and lung fibrosis, early medication focuses on atypical epithelial and fibrotic proliferation, such as Nintedanib, may be a potential therapeutic method to decrease the mortality of COVID-19. Nintedanib is an inhibitor of multiple tyrosine kinases including vascular endothelial growth factor (VEGF), platelet-derived growth factor (PDGF) and fibroblast growth factor (FGF) (33), which is approved for the treatment of progressive fibrosing interstitial lung diseases (PF-ILD) (34) in March, 2020. It was reported that infection of SARS-CoV-2 resulted in significant elevation of many cytokines such as IL-2, VEGF, PDGF and FGF (35, 36). Hence, some hypotheses based on the potential effects of antifibrotic drugs like Nintedanib on COVID-19 was proposed in a Personal View published in May, 2020 (37). As an antifibrotic drug, Nintedanib also proved to have antiproliferative activity in non-small-cell lung cancer (NSCLC) (38) and its efficiency combined with other anti-tumor drugs on NSCLC was confirmed in clinical trials (39). Moreover, Nintedanib was reported to inhibit transforming growth factor-β (TGF-β) signaling pathway by suppressing phosphorylation of Smad2/3 and type II TGF-β receptor in human lung fibroblasts (33) and A549 cells (alveolar epithelial-type II cells models) (40), which were considered as novel antifibrotic mechanisms of Nintedanib. Meanwhile, another study found the relation between expression of CEA (CEACAM5) gene and TGF-β pathway genes (TGFBR1, TGFBR2, and SMAD3) in colorectal adenocarcinoma cells (41). Though there were few publications about the association in alveolar epithelial cells, it is possible that similar mechanisms existed in human alveolar epithelial cells, especially when CEA on the cell membrane functioned as microbial receptors for bacteria or viruses. In fact, TGF-β pathway plays a role in the pathogenesis of SARS-CoV-2 (42). However, the detailed mechanisms of CEA or effects of Nintedanib remains unknown in SARS-CoV-2 infected alveolar epithelial cells. More in-depth studies of related molecular mechanisms need to be conducted in the future. Overall, considering significant elevated CEA levels originating from atypical epithelial proliferation in non-survivors with COVID-19 of our study, we think Nintedanib may be one of therapeutic options for antifibrotic therapy in addition to other basic treatment (oxygen therapy, antiviral therapy etc.) for COVID-19 patients especially those critical cases with elevation of CEA levels. It is possible that additional antifibrotic therapy might be an effective supplement for the present COVID-19 treatment strategy. More pilot studies can be performed to validate the effects of antifibrotic therapy on COVID-19 in the future.

A few limitations existed in our study. Firstly, the number of the participants was not very large relatively. The patients with COVID-19 were from the same hospital and selection bias of the patients may exist in our study. The association should be validated in another cohort with larger sample size. Secondly, the CT scores of death cases in ICU were missing because these patients were impossible to have CT examinations in view of their critical condition. Thus, we cannot analyze the CT scores in the death group with COVID-19. Finally, the complications of other organs besides lung cannot be analyzed in discharged patients with COVID-19 due to a lack of data.

In conclusion, our study suggested that the elevation of serum CEA levels increased the risk of death from COVID-19 and serum CEA levels were related to CT scores of the discharged patients positively. Serum CEA levels might be a potential biomarker to evaluate the severity and prognosis of COVID-19. More prospective and multicenter studies with validation cohorts are needed to be conducted in the future.

## Data Availability Statement

The raw data supporting the conclusions of this article will be made available by the authors, without undue reservation.

## Ethics Statement

The studies involving human participants were reviewed and approved by Ethics Committee of Wuhan Jin Yin-tan Hospital (No. KY-2020-59.01). Written informed consent for participation was not required for this study in accordance with the national legislation and the institutional requirements.

## Author Contributions

JT, YL, MH, and WX contributed to funding acquisition, project administration, and supervision. QC and HK contributed to and writing original draft. XQ and WD contributed to software application and formal analysis. CH and WW contributed to data curation and investigation. NJ and CW contributed to methodology. MH and WX contributed to reviewing and editing original draft. All authors reviewed and approved the final manuscript.

## Conflict of Interest

The authors declare that the research was conducted in the absence of any commercial or financial relationships that could be construed as a potential conflict of interest.
