# Supplementary material for: Carcinoembryonic Antigen: A Potential Biomarker to Evaluate the Severity and Prognosis of COVID-19
Source: Front Med (Lausanne). 2020 Oct 6;7:579543. doi: 10.3389/fmed.2020.579543 (PMC7573292; doi:10.3389/fmed.2020.579543)
Supplement: Supplementary file 1 [file Data_Sheet_1.docx]

**Clinical classification of COVID-19**

The clinical classification of COVID-19 in our study was based on “Diagnosis and Treatment Protocol for Novel Coronavirus Pneumonia (Trial Version 7)” published by the National Health Commission of the People’s Republic of China. Patients with COVID-19 are divided into 4 types: mild cases, moderate cases, severe cases and critical cases. Mild cases are defined as the patients who have mild clinical symptoms without any sign of pneumonia radiographically. Moderate cases are defined as patients who have respiratory symptoms and fever along with radiological sign of pneumonia. Severe cases are defined as the patients who meet any of the following criteria for adults: (1) Respiratory distress with respiratory rate > 30 times per minute. (2) Oxygen saturation no more than 93% at rest. (3) Ratio of partial pressure of arterial blood oxygen (PaO_2_) to fraction of inspired oxygen (FiO_2_) less than 300 mmHg. (4) Patients with obvious progression (more than 50% area of lung injuries than before) on radiological imaging within 24-48 hours should be treated as severe cases. Critical cases fulfil any of the following criteria: (1) Respiratory failure in need of mechanical ventilation. (2) State of shock. (3) Other organ failure with the requirement of ICU care.

**Criteria of radiological assessment by CT scores**

The radiologists who scored the images were blind to the clinical data of all patients. The whole lung was divided into three regions including upper region (above the carina), middle region (between the carina and the inferior pulmonary vein) and lower region (below the inferior pulmonary vein). A total of six regions required to be included with the left and right lung scored separately. The criteria for the CT scores of each region were as follows: 0 points for no lung lesions; 1 point for less than 25% lung lesions; 2 points for 25%-50% lung lesions; 3 points for 50%-75% lung lesions; 4 points for over 75% lung lesions. The total CT points were calculated by adding up each score of the six lung regions and the final scores of each patient were the average CT points scored by two radiologists. The following abnormalities were considered as lung lesions: thicken bronchial wall, bronchiectasis, ground-glass opacities, lung consolidation or effusion, formation of cavities or honeycombs, enlarged lymph nodes or vessels and
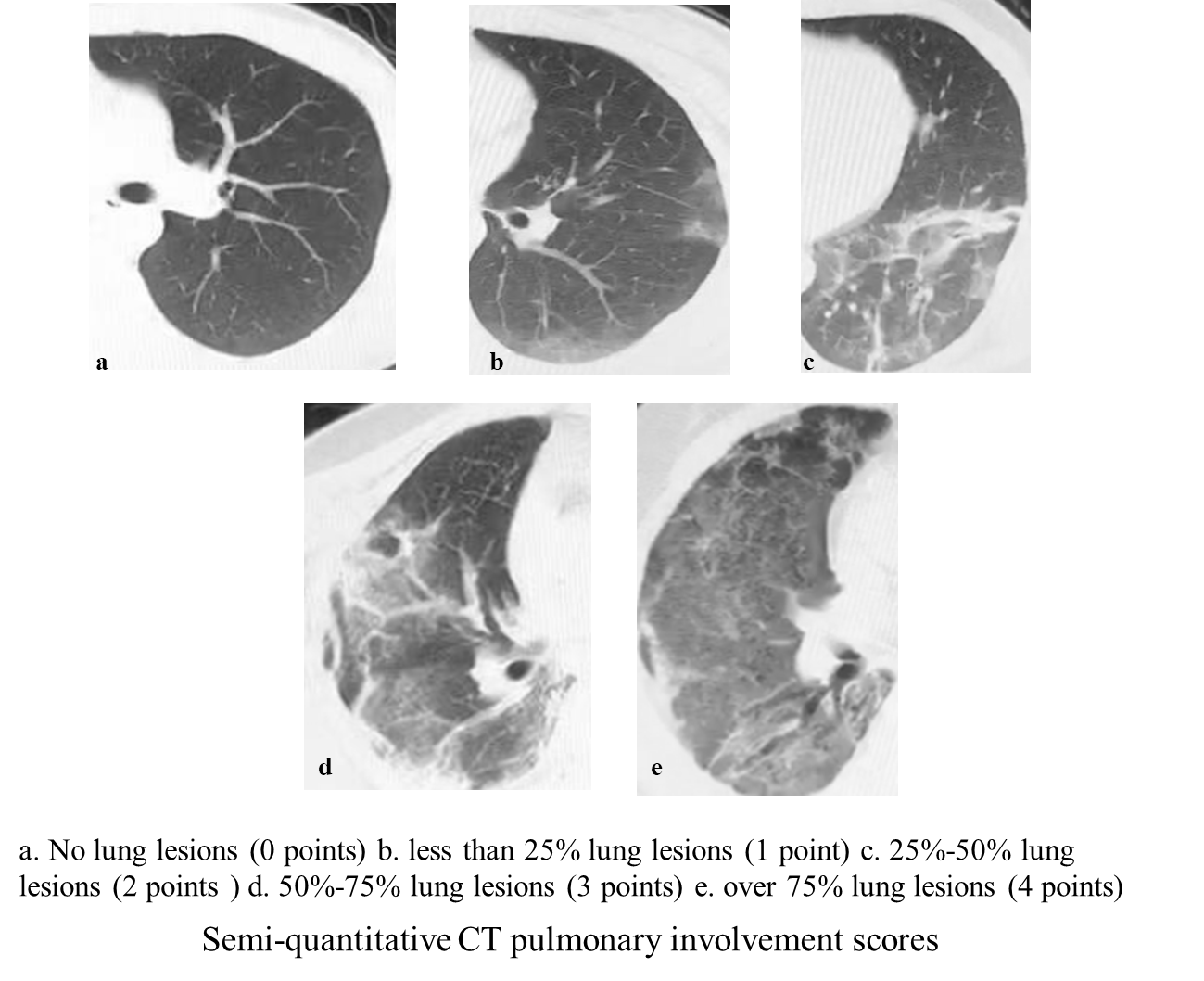
structural destruction.
